# Supplementary material for: A high-resolution mRNA expression time course of embryonic development in zebrafish
Source: eLife. 2017 Nov 16;6:e30860. doi: 10.7554/eLife.30860 (PMC5690287; doi:10.7554/eLife.30860)
Supplement: Supplementary file 6. [file elife-30860-supp6.zip › biolayout-clusters-files/Cluster249-genes.html]

Cluster249


# Cluster249: Genes

| Ensembl ID | Gene Name | Chr | Start | End | Biotype |
| --- | --- | --- | --- | --- | --- |
| ENSDARG00000058232 | fbrsl1 | 5 | 17165650 | 17249819 | protein\_coding |
| ENSDARG00000031048 | gspt1 | 12 | 19240461 | 19262071 | protein\_coding |
| ENSDARG00000060397 | hhip | 1 | 35058055 | 35103836 | protein\_coding |
| ENSDARG00000007396 | rnd3b | 9 | 23215455 | 23222949 | protein\_coding |
| ENSDARG00000033916 | si:ch211-243j20.2 | 20 | 38717787 | 38767817 | protein\_coding |
| ENSDARG00000074502 | zmiz1a | 13 | 30291022 | 30440409 | protein\_coding |
